# Mantle-derived helium released through the Japan trench bend-faults

**DOI:** 10.1038/s41598-021-91523-6

**Published:** 2021-06-14

**Authors:** Jin-Oh Park, Naoto Takahata, Ehsan Jamali Hondori, Asuka Yamaguchi, Takanori Kagoshima, Tetsuro Tsuru, Gou Fujie, Yue Sun, Juichiro Ashi, Makoto Yamano, Yuji Sano

**Affiliations:** 1 Atmosphere and Ocean Research Institute, University of Tokyo, Kashiwa, Japan; 2 Department of Environmental Biology and Chemistry, University of Toyama, Toyama, Japan; 3 Department of Marine Resources and Energy, Tokyo University of Marine Science and Technology, Tokyo, Japan; 4 Japan Agency for Marine-Earth Science and Technology, Yokohama, Japan; 5 Earthquake Research Institute, University of Tokyo, Tokyo, Japan; 6 Center for Advanced Marine Core Research, Kochi University, Nankoku, Japan

**Keywords:** Ocean sciences, Solid Earth sciences

## Abstract

Plate bending-related normal faults (i.e. bend-faults) develop at the outer trench-slope of the oceanic plate incoming into the subduction zone. Numerous geophysical studies and numerical simulations suggest that bend-faults play a key role by providing pathways for seawater to flow into the oceanic crust and the upper mantle, thereby promoting hydration of the oceanic plate. However, deep penetration of seawater along bend-faults remains controversial because fluids that have percolated down into the mantle are difficult to detect. This report presents anomalously high helium isotope (^3^He/^4^He) ratios in sediment pore water and seismic reflection data which suggest fluid infiltration into the upper mantle and subsequent outflow through bend-faults across the outer slope of the Japan trench. The ^3^He/^4^He and ^4^He/^20^Ne ratios at sites near-trench bend-faults, which are close to the isotopic ratios of bottom seawater, are almost constant with depth, supporting local seawater inflow. Our findings provide the first reported evidence for a potentially large-scale active hydrothermal circulation system through bend-faults across the Moho (crust-mantle boundary) in and out of the oceanic lithospheric mantle.

## Introduction

Water transported by subducting oceanic plates from the Earth’s surface into its interior has been widely recognized. Hydration of the oceanic plate prior to subduction constrains the amount of water transported^1,2^. Plate bending-related normal faults (i.e. bend-faults) develop at the outer trench-slope of the oceanic plate incoming into the subduction zone^3–5^. During the past decades, extensive seismic, electromagnetic, and heat flow surveys conducted at Japanese, Alaskan, Central American, and South American subduction zones have reported the presence of a subseafloor water-bearing layer^5–16^. All the cited studies^5–16^ proposed that persistent brittle bend-faulting across the entire outer trench-slope might provide high-permeability pathways for intensive fluid transport into the lithosphere, which can be driven by extension associated with plate bending, thereby promoting hydration of the oceanic plate. However, the buoyancy of water (or equivalently, confining pressure) makes it difficult to bring seawater down even if bend-faulting reaches into the mantle. Extension associated with plate bending generates negative dynamic pressure, but the magnitude of such dynamic pressure might be insufficient to overcome confining pressure^17^, leaving the depth of seawater penetration debated.

**Figure 1:**
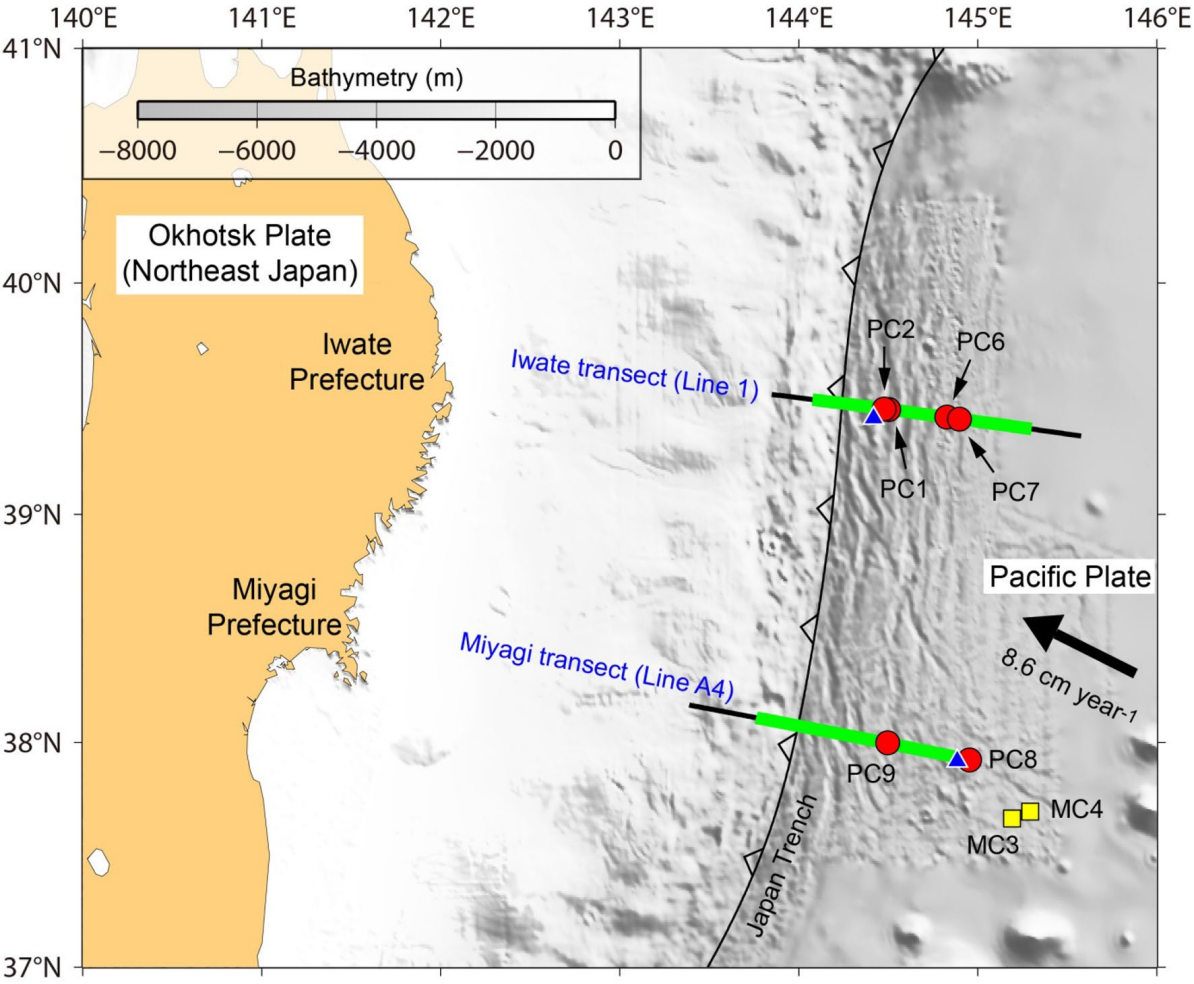
Bathymetry map of the Japan trench margin offshore NE Japan. The Pacific plate subducts beneath the Okhotsk plate (NE Japan) at a convergence rate of 8.6 cm year^−1^ (ref.^20^). Heavy green lines mark MCS profiles depicted in Fig. 3, which are on the two lines of A4 (Miyagi transect) and 1 (Iwate transect). Six red circles (PC1 to PC9) on the two MCS lines indicate the gravity coring sites. Two yellow squares (MC3 and MC4) indicate the bottom seawater sampling sites. Two blue triangles mark petit-spot volcanoes^21,22^ nearest from lines A4 and 1, respectively.

The Japan trench margin (Fig. 1), offshore of north-eastern Japan, is a well-suited natural laboratory for the study of the bend-faults and their association with oceanic plate hydration^3,4,13,14,18^. For example, hydration of the oceanic crust and upper mantle is suggested by the reduction of seismic velocities and anomalously high *V*_P_/*V*_S_ ratios^13,14^. Despite the significance of the bend-faults at the incoming Pacific plate, few observations have elucidated the fault characteristics and potential fluid flow along the faults. To examine the geometry and associated fluid flow of Japan trench bend-faults, a seismic reflection survey and a geochemical observation of pore fluids were carried out in 2019, specifically using the helium isotope ratio as a valuable tracer for identifying fluid origins^19^. This study reports mantle-derived helium released through the Japan trench bend-faults and propose a hydrothermal circulation through bend-faults across the Moho.

## Results and discussion

### Anomalous ^3^He/^4^He ratios near the bend-faults

**Figure 2:**
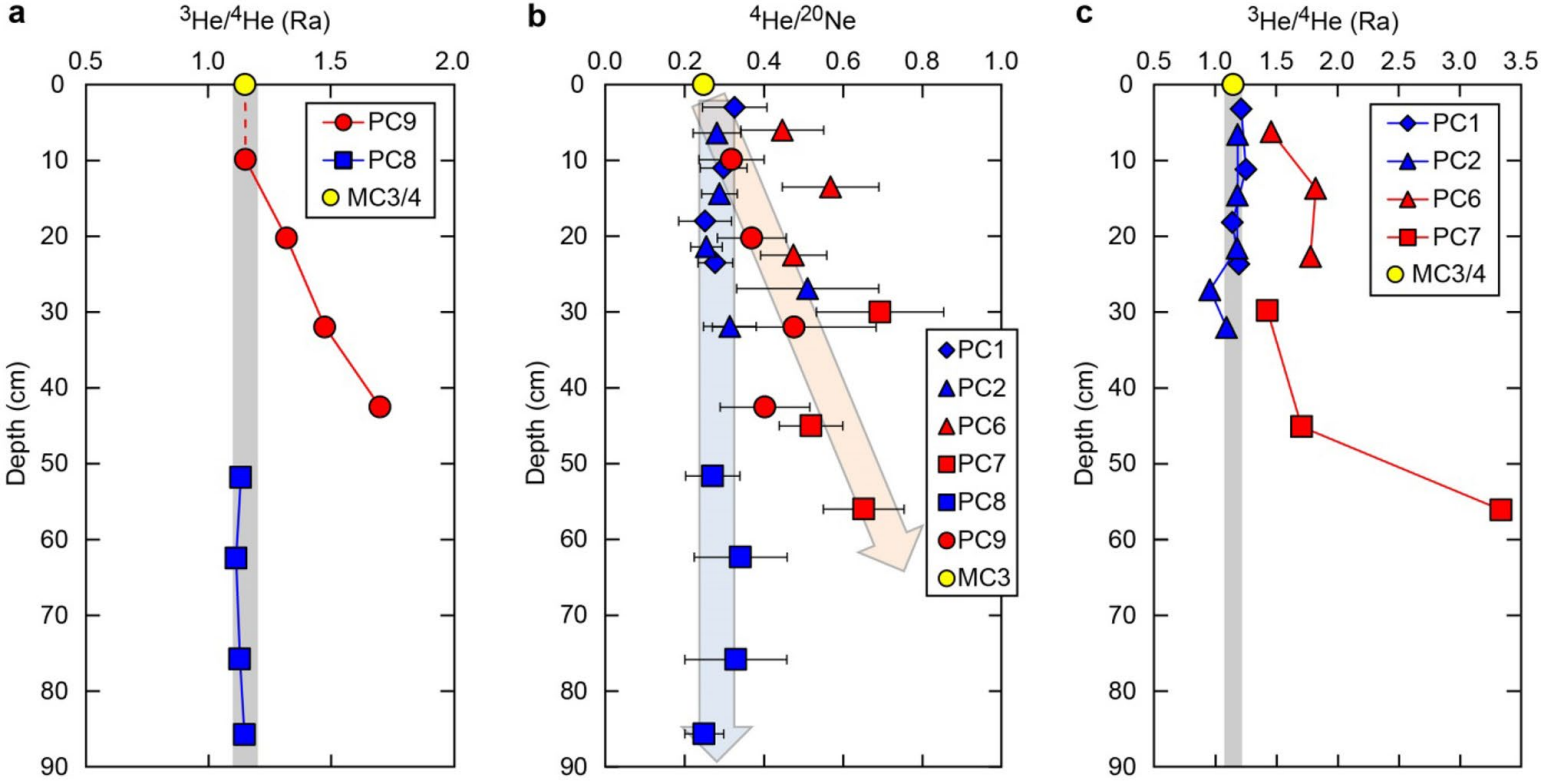
Depth profiles of ^3^He/^4^He and ^4^He/^20^Ne ratios at sites PC1–PC9, MC3, and MC4. (**a**) ^3^He/^4^He ratios on the Miyagi transect (line A4). Vertical gray zone marks the ^3^He/^4^He ratio of ca. 1.15 Ra, which represents average northwest Pacific bottom seawater^29^. The helium isotopes ratios at site PC9 increase with depth, whereas those at site PC8 are almost constant at ca. 1.15 Ra. (**b**) The ^4^He/^20^Ne ratios on the Miyagi and Iwate transects. The ratios at sites PC6, PC7, and PC9 increase with depth, whereas those at sites PC1, PC2, and PC8 are nearly constant with depth. (**c**) The ^3^He/^4^He ratios on the Iwate transect (line 1). Ratios at sites PC6 and PC7 increase with depth, whereas those at sites PC1 and PC2 are almost all constant along the value of ca. 1.15 Ra.

At two transects crossing the Japan trench, surface sediments were collected by using gravity corers at six seafloor sites located nearby the bend-faults, and then pore-fluids were extracted from the surface sediment samples (Fig. 1, “Methods” section). Below those sites, reduced seismic velocities (about 10% *V*_P_ reduction)—suggesting a hydrated oceanic crust and upper mantle—were clearly observed to about 100 km seaward from the trench^13,23^. In pore-water samples at three sites of PC9, PC6, and PC7 (Figs. 1, 2), helium isotope ratios (^3^He/^4^He or R, normalized to air ratio Ra = 1.38 × 10^–6^; ref.^24^) higher than the expected value of seawater (i.e. ca. 1 Ra; ref.^25^) suggests the presence of mantle-derived helium. The ^3^He/^4^He ratios higher than crustal (0.02 Ra) or atmospheric (1 Ra) sources, originated from the addition of mantle helium, are usually found close to active volcanic areas and oceanic ridges: some examples are the Juan de Fuca Ridge and the East Pacific Rise^26–28^. Our findings constitute the first report of mantle-derived helium observed in pore water from sediments of the oceanic plate incoming to a deep-sea trench.

On the transect offshore Miyagi prefecture (hereafter the Miyagi transect, Fig. 1), the ^3^He/^4^He ratio at site PC9 increases distinctly with sediment depth below seafloor (i.e., 1.15–1.70 Ra; Fig. 2a, Supplementary Table S1). On the other hand, the ratio at site PC8 is almost constant around 1.11–1.15 Ra (Fig. 2a). The high ^3^He/^4^He ratio of 1.70 Ra at site PC9 is remarkably higher than the average value of approx. 1.15 Ra reported for northwest Pacific bottom seawater^29^, which is also verified from bottom seawater samples at sites MC3 and MC4 (Figs. 1, 2a, Supplementary Table S1). The increasing ^3^He/^4^He ratio with sediment depth at site PC9 suggests a vertical flux of mantle fluids containing ^3^He, supplied toward the seafloor, as observed in the Okinawa Trough backarc basin^30^. The ^4^He/^20^Ne ratio (Fig. 2b, Supplementary Table S1) reflecting the helium concentration at site PC9 increases with depth, suggesting an upward flux of helium with the deep source.

**Figure 3:**
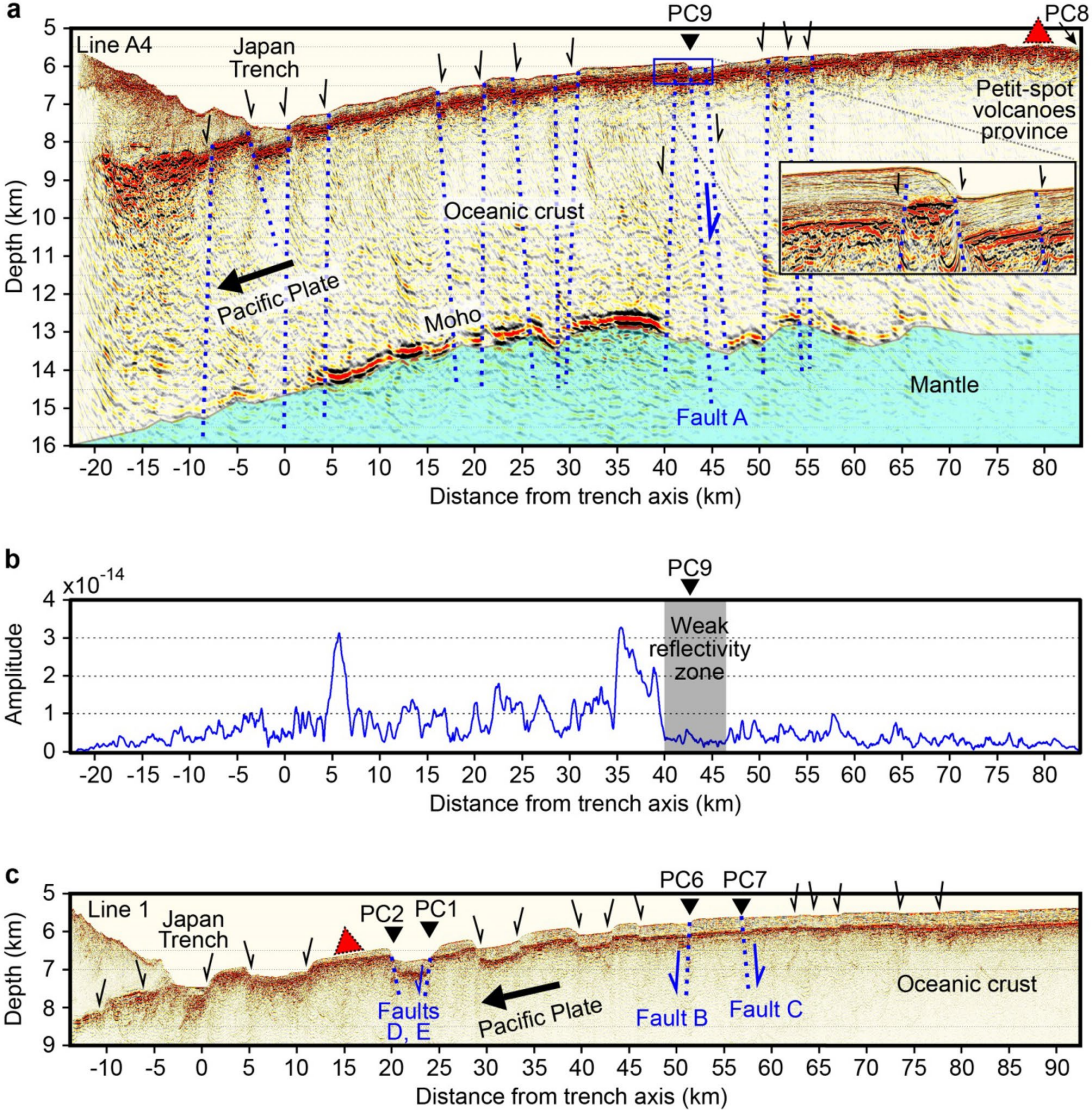
Seismic reflection data on lines A4 (Miyagi transect) and 1 (Iwate transect). Gravity coring sites of PC1–PC9 are shown. Two petit-spot volcanoes^21,22^ marked by red triangles, which are the nearest from lines A4 and 1, are projected to each line. (**a**) RTM depth section with automatic gain control (AGC) on line A4. Vertical exaggeration (VE) is 4:1. We identify westward and eastward dipping normal faults. Inset: High-resolution MCS section (“Methods” section), focusing on fault A and its neighbouring faults near site PC9, is shown. (**b**) Root mean square (RMS) amplitudes without AGC of the Moho reflection on the RTM result of line A4. A weak reflectivity zone of the Moho is apparent at approx. 40–46 km distance. (**c**) K-PSDM section with AGC on line 1. VE is 4:1. We identify many normal faults, producing horst-and-graben structures.

The multi-channel seismic (MCS) depth section developed by applying reverse time migration (RTM) on line A4 (Fig. 3a, “Methods” section; see Supplementary Figs. S1a, S2a and S3 for uninterpreted profile, interval velocity model, and velocity uncertainty test, respectively) along the Miyagi transect reveal a normal fault “A” near site PC9, which is interpreted to develop from the seafloor down to the upper mantle through the Moho at approx. 13 km depth. The steeply eastward-dipping (approx. 74° dip in the shallow section) bend-fault A exhibits a similar fault offset (approx. 180 m) at the seafloor and the Moho depth. This implies that the fault across the entire oceanic crust (approx. 7 km thick) was recently formed at the outer trench-slope. The fault dip angle of approx. 74° is measured by the offset from the seafloor to the top of the igneous basement (i.e. the oceanic crust), and thus could be somewhat overestimated because of possible seafloor sediment reworking such as slumping and resedimentation. Most of fault planes, derived from extensional earthquakes with normal-faulting focal mechanisms at the outer rise, are 50°–75° for the Japan trench^31^ and 45°–55° for the Middle America and Chile trenches^32^. For that reason, the steep dip of the bend-fault A appears to be limited to the seafloor sediment section, and the fault is likely to evolve to lower dip angles as extension progresses deeper. The Moho below site PC9 shows remarkably weak reflectivity over a region of 40–46 km horizontal distance (Figs. 3a, b). Root mean square (RMS) amplitude averaged over the 6-km-wide, weak reflectivity zone, is approximately 1.4 and 5 times lower than those of neighbouring regions (i.e. 46–65 km and 30–40 km distances, respectively), indicating seismic attenuation such as seismic wave scattering or absorption. This result suggests the occurrence of a large-scale fractured zone around the Moho, which could result from persistent brittle faulting breaking through the oceanic crust into the upper mantle. Neighbouring faults close to the fault A, imaged on the high-resolution MCS profile (inset of Fig. 3a; see Supplementary Fig. S1a for uninterpreted profile), might help to cause the 6-km-wide fractured zone. Such a fault-related fractured zone is likely to provide high-permeability pathways for fluids reaching the mantle. We observe similar weak reflectivity of the Moho at the deep extension of the shallow normal fault (for example, at 0 km, 18 km, 22 km, and 29 km distances in Fig. 3a), which may also be caused by persistent brittle faulting. The RMS amplitude averaged over a region of 65–84 km distance, which is characterized by thin sediment on the igneous basement, is approximately 23% lower than that of the weak reflectivity zone (40–46 km distance), probably because of considerable seismic attenuation by petit-spot magmatism originated from partial melts along lithospheric fractures in response to plate flexure during subduction^22^.

High ^3^He/^4^He ratios have been observed in groundwater close to the San Andreas Fault (maximum 4.0 Ra) in California^33^ and the Yangsan Fault (maximum 5.66 Ra) of the southeastern Korean Peninsula^34^, and in bottom seawater close to the Marmara Main Fault (maximum 4.9 Ra) in Turkey^35^, indicating mantle degassing along these seismically active, crustal-scale faults. Similarly, faulting-related characteristics below site PC9, which shows a high ^3^He/^4^He ratio indicative of addition of mantle-derived fluids, enable us to infer that bend-fault A might function as a pathway for the mantle fluids to migrate into the oceanic crust toward the seafloor.

On the transect offshore Iwate prefecture (hereafter the Iwate transect), the ^3^He/^4^He ratios at sites PC6 and PC7 increase with sediment depth below seafloor (1.46–1.78 Ra and 1.43–3.34 Ra, respectively) (Fig. 2c), indicating a significant helium input from the mantle. By contrast, the ^3^He/^4^He ratios at sites PC1 and PC2 are nearly constant with depth (1.14–1.25 Ra and 0.96–1.19 Ra, respectively) and close to the value of ambient seawater^29^, suggesting only a minimal input of mantle-derived helium.

The Kirchhoff pre-stack depth migration (K-PSDM) profile of line 1 (Fig. 3c, Supplementary Fig. S4a presents an uninterpreted profile) along the Iwate transect reveals steeply westward-dipping and eastward-dipping normal faults “B” and “C”, respectively, near sites PC6 and PC7. The fault B shows a similar fault offset (approx. 100 m) at the seafloor and the top of the igneous basement, and the fault C shows a similar fault offset (approx. 40 m) at those interfaces, suggesting that these faults are recently formed. Faults B and C also might play a role as pathways for the mantle fluids to migrate up to the seafloor. However, it remains unclear whether those faults reach into the upper mantle because of insufficient penetration of air-gun sources used in acquiring these MCS data. The MCS profile of line 1 also exhibits normal faults “D” and “E” with fault offsets of approx. 450 m and approx. 350 m, respectively, at the top of the igneous basement of sites PC2 and PC1.

### Active hydrothermal circulation across the Moho

The linkage between geochemical and geophysical observations at sites PC6, PC7, and PC9 on the incoming Pacific plate supports the outflow of mantle fluids through the bend-faults as we discussed above. To explain the anomalously high helium isotope (^3^He/^4^He) ratios near the bend-faults, in addition to supply of mantle-derived helium through the bend-faults, two alternative scenarios can be taken into account: (1) a contamination of mantle helium from petit-spot volcanoes^21,22^ discovered at several sites seaward of the Japan trench; (2) cosmic dust in sediments with a high ^3^He/^4^He ratio contributed to the high ^3^He (ref.^36^).

The petit-spot magmatism is believed to have originated from melts along lithospheric fractures in response to plate flexure during subduction: therefore, the subducting plate is continuously subject to the magmatism as it bends at the outer rise. Anomalously high temperature data of mantle xenoliths recovered from petit-spot basalts suggest that the total melt volume delivered from the asthenosphere would be greater than that of the surface eruption by 2–3 orders of magnitude^37^, implying that the excess ^3^He at three sites (PC6, PC7, and PC9) within approx. 40 km distance from the nearest petit-spot volcano may be associated with the petit-spot magmatism. However, we note a roughly inverse relation between the distance from the nearest petit-spot volcano and the ^3^He/^4^He ratios in sediment pore water (Supplementary Fig. S5). Sites PC1, PC2, and PC8 closer to the volcanoes, which are one (location: 39° 25′ N, 144° 25′ E) within Site A region^21^ and another (location: 37° 55′ N, 144° 53′ E) within Site C region^22^, show an average ^3^He/^4^He ratio of 1.15 Ra close to values from bottom seawater samples. This suggests that the petit-spot volcanoes do not essentially add excess ^3^He at sites PC6, PC7, and PC9 which are more than 35 km away from the nearest petit-spot volcanoes. It is similar to a case of Japanese land volcano, which is that excess ^3^He at sites beyond approx. 30 km away from the land volcano has been rarely observed^38^. On the other hand, it is different from cases of Italian land volcanoes; for instance at Mt. Etna volcano, mantle helium signatures were observed at sites beyond about 40 km away from the volcano^39^. Despite a sporadic nature of petit-spot volcanism at the outer rise^22,37^, such a roughly inverse relation between the location of petit-spot volcanoes and mantle helium signatures is not compatible with an addition of mantle helium from the petit spot volcanoes at sites PC6, PC7, and PC9. In fact, although a petit-spot volcano is often invoked as “the tip of the iceberg”^37^, there have been few geophysical observations such as magnetic anomalies to support the existence of the iceberg under sites PC6, PC7, and PC9.

One might argue that cosmic dust in sediments with a high ^3^He/^4^He ratio contributed to the high ^3^He (ref.^36^). However, we measured the sedimentary phase at the sampling sites (“Methods” section), which revealed that the helium isotopic ratio is low, with values of 0.17–0.29 Ra. The ^4^He/^20^Ne ratio (Fig. 2b, Supplementary Table S1) reflecting the helium concentration^30^ at site PC9 increases with depth, suggesting an upward flux of helium with the deep source. Perhaps mantle helium, as a volatile, could rise through the oceanic crust via diffusion and only become entrained in pore fluid within the upper crust. However, it seems very difficult that mantle helium rises through the oceanic crust via diffusion, because the diffusion speed is too slow (for example, the molecular diffusion coefficient of helium is 4.2 × 10^−5^ cm^2^ s^−1^ at seawater temperature^40^) for mantle helium to be observed at the ocean floor surface. That is why it is generally thought that mantle helium is completely isolated from the normal ocean floor (without tectonic activities such as volcanic eruptions) on which mantle helium has not been observed^41^. Volcanism would be a major route to carry such a mantle helium to Earth’s surface. Deep fluid flow as a carrier would be required for mantle helium to be observed on the surface. We conclude, therefore, that the bend-faults as fluid conduits extending into the upper mantle are more plausible to explain the abnormal ^3^He/^4^He ratio at sites PC6, PC7, and PC9, even though we cannot completely rule out the impact of petit-spot volcanoes.

**Figure 4:**
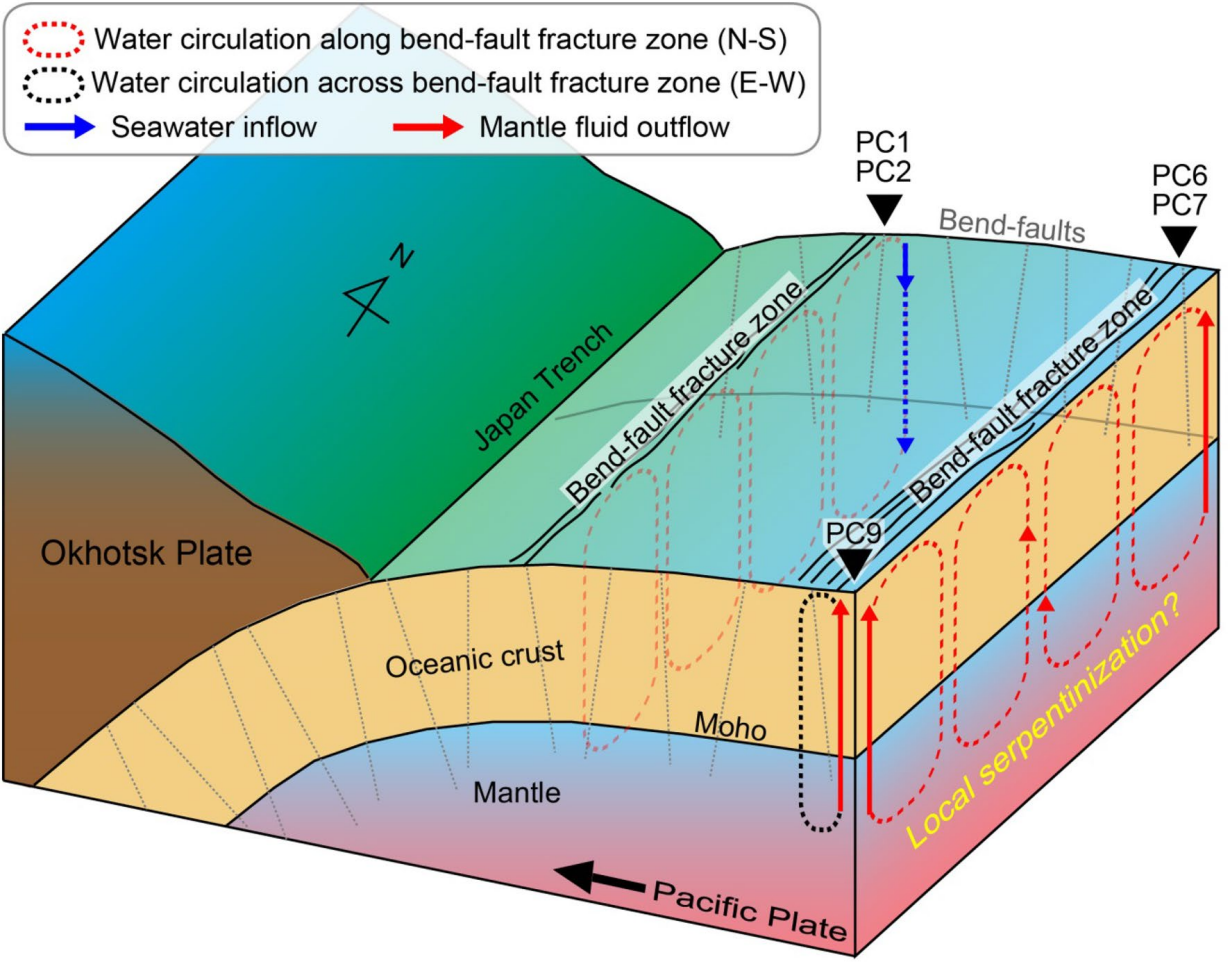
Schematic drawing of hydrothermal circulation across the Moho in the outer slope of the Japan trench. The water circulation across the Moho, which might be driven by thermal buoyancy, would occur along the same bend-fault fracture zone, that is, north–south parallel to the trench (i.e. red dashed hydrothermal convection cell). Structural continuity of the bend-fault fracture zone between sites PC6 and PC9 is not known yet. If the bend-fault fracture zone with high permeability is sufficiently wide, water circulation across the bend-fault fracture zone (that is, east–west) can also occur within the wide fracture zone (i.e. black dashed hydrothermal convection cell). Limited serpentinization may occur in the uppermost mantle.

When we consider a conceptual model of fluid circulation (Fig. 4), the mantle fluid outflow requires the existence of the seawater penetrated down to mantle depths (i.e. the seawater inflow) because the uppermost mantle is usually inferred to be essentially dry^42^, even though there is no direct observation that the seawater reaches the uppermost mantle. In other regions of the forearc region of the Japan trench^43^, the ^3^He/^4^He ratios in pore water not affected by mantle fluids decrease considerably with depth because of the addition of radiogenic ^4^He produced within the crust from U and Th decay. However, ^3^He/^4^He ratios at sites PC1 and PC2 near the trench (Figs. 2c, 3c) on the Iwate transect (line 1) are almost constant with depth and are close to the typical value of 1.15 Ra for northwest Pacific bottom seawater, suggesting a persistent supply of seawater into the bend-faults near sites PC1 and PC2. Nearly constant ^4^He/^20^Ne ratios (Fig. 2b) with depth at sites PC1 and PC2, which are close to the isotope ratio of bottom seawater, also favour the seawater inflow. It remains unclear whether site PC8 with similar ^3^He/^4^He and ^4^He/^20^Ne ratios to those of sites PC1 and PC2 (Figs. 2a, b) could be a candidate for seawater inflow down to mantle depths, because of no seismic image of bend-faults in the vicinity of the site. One might argue that isotopic values from these ~ 24- to 86-cm-long gravity cores at sites PC1, PC2, and PC8 simply reflect that the pore fluids of the seafloor sediment are equilibrated with seawater. Indeed, there are several studies in the Okinawa Trough^30^, forearc region of the Japan trench^43^, and the Nankai Trough^44^ reporting that pore fluids at depths of several tens of centimetres are not equilibrated with seawater, implying that those isotopic values at sites PC1, PC2, and PC8 also are not likely to be equilibrated with seawater.

Seawater, transported down to mantle depths, usually reacts with mantle peridotite surrounding the bend-fault, leading to mantle serpentinization^5^ suggested by seismic velocity reductions^13,14^ whose interpretation is still a matter of debate. In fact, an increase in water-filled cracks^17^ as well as serpentinization can explain the seismic velocity reductions with smaller amounts of water. A recent study on seismic anisotropy measurements shows that upper mantle hydration at the Middle America trench (MAT) is limited to serpentinization and/or water in fault zones^45^. If the Japan trench is the similar case to the MAT, only a small amount of the seawater reaching the uppermost mantle and potentially stored in the form of pore fluids would be consumed by the serpentinization reactions, implying that some of the remainder may outflow by the fluid circulation.

Widespread, anomalously high heat flow in the incoming Pacific plate imply hydrothermal circulation in a permeable layer within the oceanic crust^18^. The hydrothermal circulation and alteration at the mid-ocean spreading ridge are also limited to the upper 2–3 km of the oceanic crust^5^. In contrast, the potential mantle fluid outflow (PC6, PC7, and PC9) and seawater inflow (PC1 and PC2) demonstrate water circulation through bend-faults in the oceanic crust and upper mantle (Fig. 4). The water circulation across the Moho, which might be driven by thermal buoyancy^46^ (i.e. hydrothermal circulation), would occur along the same bend-fault fracture zone (i.e. north–south parallel to the trench). If the oceanic crust and the uppermost mantle are severely fractured by brittle faulting, their permeabilities would be sufficiently high to enable such a hydrothermal circulation along the fracture zones of the oceanic crust and the uppermost mantle. Upward fluid flows along the hydrothermal convection cells (Fig. 4) might be observed at three sites (PC6, PC7, and PC9) showing excess ^3^He. In contrast, another two sites (PC1 and PC2) suggesting a supply of seawater into the faults, can be associated with downward flows along the convection cells. Additionally, if the 6-km-wide weak reflectivity zone (Fig. 3a) around the Moho below site PC9 corresponds to the bend-fault fracture zone with high permeability, water circulation east–west across the bend-fault fracture zone can occur within the fracture zone (i.e. 6-km-wide convection cell).

Despite a scale gap between pore-fluids sampling depth (less than 1 m) and the Moho depth ca. 7 km below seafloor, it is proposed that the bend-faults at the outer slope of the Japan trench enable a large-scale, active hydrothermal circulation across the Moho in the oceanic lithosphere consisting mainly of the oceanic crust and the uppermost mantle. As a future study, additional deep seismic imaging of bend-faults across the Moho and detailed geochemical observation of other mantle volatiles such as CO_2_ are required to properly assess the generality of our interpretation on Miyagi and Iwate transects. Time-series geochemical observations at additional sites also would constrain detailed fluid flux and the water circulation pattern. Ocean drilling of the bend-faults, which enables us to define mechanical and hydrological properties of the faults and to observe the mantle fluids at deeper depths, would be useful for more comprehensive understanding of the mantle fluid flow.

## Methods

### Noble gas sampling and analysis

To elucidate fluid origins at the outer trench-slope of the Japan trench, we collected sediment samples from six seafloor sites during the KS-19-14 cruise of R/V *Shinsei-Maru* of July–August 2019 (Supplementary Table S1). To minimize seawater contamination caused by coring operations, we only used samples that were collected by gravity corers (a G.S.-type triple-tube core sampler and a gravity corer) for geochemical analysis, which are usually used as pilot weights of a piston corer system. A sampling method developed by Pitre and Pinti (ref.^47^) was applied to these sites. The samples were transferred immediately into copper tubes. Both ends were sealed by metal clamps on the ship to avoid air contamination. At the Atmosphere and Ocean Research Institute, helium and neon gases dissolved within the pore fluid samples were extracted from centrifuged sediment samples; then they were introduced into the vacuum line connected to a quadrupole mass spectrometer (QMS; Pfeiffer Prisma 80) and a high-precision, noble gas mass spectrometer (Helix SFT; GV Instruments Ltd.)^19^. In the line, the sample gases were purified of reactive gases. Then the ^4^He/^20^Ne ratios were measured using the QMS. After the helium was separated from the neon by a charcoal trap held at 40 K, the ^3^He/^4^He ratios were measured using the Helix SFT and were calibrated against those of the atmosphere standard. In a similar manner, we measured the ratios of ^4^He/^20^Ne and ^3^He/^4^He for bottom seawater samples collected from two sites by the multiple corer system during the KS-19-13 cruise of R/V *Shinsei-Maru* in July 2019 (Supplementary Table S1). To measure helium in the solid sediment, we used a dried sediment that remained in pore fluid samples after helium analysis. The helium in the solid sediment was extracted at temperatures of 1800 °C by heating in a furnace. After the extraction, the analysis procedure for helium and neon isotopes is the same as that for the pore fluid sample.

The experimental errors of the ^3^He/^4^He ratio and ^4^He/^20^Ne ratio were about 4% and 20% (2σ), respectively, estimated by repeated measurements of standard air containing concentrations equivalent to those of the samples and analytical error of each measurement of a water sample. As an additional check on the accuracy of the analytical system, we analyzed air-equilibrated seawater samples taken from a water reservoir in a thermostatically controlled room. The observed data agreed well with values reported in a literature^48^ within the experimental error. The helium blank level (the same experimental procedure without the sample) was less than 10% of the level of the samples, and the blank ^3^He/^4^He ratio was atmospheric within analytical error, therefore negligibly affected the calibrated ratios.

### Seismic data acquisition, processing and uncertainty estimation of seismic velocities

On the Miyagi transect (line A4), multi-channel seismic (MCS) reflection data were acquired by R/V *Kairei* in February 2017. For deep-penetration seismic imaging, a large air-gun array (total volume approx. 128 L) was used as the controlled sound source. The MCS data were collected with 50 m shot interval and recorded with an approx. 6000 m, 444-channel streamer with 12.5 m group spacing. We applied conventional MCS data processing techniques including trace editing, band-pass filtering (3-6-100-125 Hz), spherical divergence correction, signature deconvolution, and common midpoint (CMP) sorting to obtain pre-conditioned CMP gathers for which the relative amplitudes are preserved. Using the CMP gathers, we developed the P-wave interval velocity models with a layer stripping method and Kirchhoff pre-stack depth migration (K-PSDM) velocity analysis^49^. Ocean-bottom seismograph (OBS) velocity data^23^ on line A4 guided the K-PSDM velocity model. We checked the final K-PSDM velocity model (Supplementary Fig. S2a) using K-PSDM gathers and their semblance plots at several distances (Supplementary Fig. S2b–g).

To estimate the uncertainty of the final K-PSDM velocity model, we performed a migration velocity scan analysis to check the flatness of the K-PSDM gather reflections for various velocity ranges (Supplementary Fig. S3). Beyond 90% and 110% of the original velocities, the Moho reflections apparently lose the flatness on the K-PSDM gathers. Their semblance maxima (blue in semblance plot) deviate from the vertical zero line (i.e., no residual depth moveout). Consequently, the velocity model has about 10% uncertainty at ca. 12 km depth. The final interval velocity model was unable to verify the reduced seismic velocities (*V*_P_) of the oceanic crust and upper mantle^23^, which were revealed by the OBS velocity data on the same line A4. The reason for that inability to verify them is that the near-vertical MCS data have a large velocity-uncertainty of about 10% because of the limited streamer cable length (approx. 6000 m long) used for deep targets and the low number of clear reflections which can be used for the K-PSDM velocity analysis.

The reverse time migration (RTM) technique, handling downward and upward wave propagations through a velocity model into the Earth, has been useful for accurate focusing and imaging of faults with a steep dip angle^50^. To obtain more detailed geologic depth image of the incoming oceanic plate, we applied RTM (Fig. 3a, Supplementary Fig. S1) at frequencies of 5–50 Hz for the pre-conditioned shot gathers using the K-PSDM velocity model (Supplementary Fig. S2a). Amplitudes of the RTM result without application of any automatic gain control (AGC) provide a fair measure to compare the seismic reflectivity. To evaluate the lateral variation of the Moho reflection amplitudes, we calculated root mean square (RMS) amplitudes with no AGC (Fig. 3b) within a gate length window of 800 m (i.e. 400 m above and below the Moho reflection) along the Moho horizon picked on the RTM profile (Supplementary Fig. S1b).

On the Iwate (line 1) and Miyagi (line 5 on the same line A4) transects, we acquired high-resolution MCS data during the KS-19-5 cruise of R/V *Shinsei-Maru* in April 2019. For the high-resolution seismic imaging, two GI guns (total volume 12 L) were used as controlled sound source. The MCS data were collected with 37.5 m shot intervals and recorded with a ca. 2000 m, 288-channel streamer with 6.25 m group spacing. We applied conventional pre-conditioning processing and K-PSDM, eventually producing detailed geologic depth profiles (inset of Fig. 3a for line 5; Fig. 3c, Supplementary Fig. S4a for line 1) and a P-wave velocity model (Supplementary Fig. S4b for line 1). We checked the final P-wave velocity model using K-PSDM gathers and their semblance plots at several distances (Supplementary Fig. S4c–e).

## Supplementary Information

Supplementary Information.
