# Supplementary material for: Mantle-derived helium released through the Japan trench bend-faults
Source: Sci Rep. 2021 Jun 14;11:12026. doi: 10.1038/s41598-021-91523-6 (PMC8203651; doi:10.1038/s41598-021-91523-6)

Supplementary Information for

**Mantle-derived helium released through the Japan Trench bend-faults**

Jin-Oh Park*, Naoto Takahata, Ehsan Jamali Hondori, Asuka Yamaguchi, Takanori Kagoshima, Tetsuro Tsuru, Gou Fujie, Yue Sun, Juichiro Ashi, Makoto Yamano, Yuji Sano

*Corresponding author. Email: jopark@aori.u-tokyo.ac.jp

**This Word file includes:**

Additional description of noble gas sampling

Supplementary Table 1

Supplementary Figures 1 to 4

**Additional description of noble gas sampling**

Here we explain how the sediment samples were transferred from the core to copper tubes without disrupting the sediment layering or allowing the noble gases to escape. We used a specially designed device which could help us take pore fluid samples without exposure to atmosphere during recovering. At sites PC1 and PC2, the liner made of acrylic, with length of 2 feet, and the outer diameter of 4 1/8” was used with the G.S.-type triple-tube core sampler. The thickness is longer than that of a normal acrylic liner used for the multiple corer. Holes matched with 1/4” ISO-brass plugs are drilled every 5-8 cm on the tube for attaching to copper tubes. All ISO-plugs locked on the tube threads of copper tubes need to be worn Teflon seal tapes ensuring the whole system is airtight. At sites PC6, PC7, PC8 and PC9, the liner made of PVC, with length of ~1 m, and the outer diameter of 80 mm was used with the gravity corer. The thickness of the liner is 3 mm.

After a core arriving onto the ship deck, two pistons are set in both sides of the tube. The top one is fixed by core holder and is designed with a valve to release water and pressure while squeezing. Once the core is ready for the next step, we can set a jack under the lower piston, attach copper tubes to the liner, and start to squeeze sediments to copper tubes. After removing water from the tube from the top valve first, and then we remove ISO-brass plugs from the acrylic liner (sites PC1 and PC2), or drill holes on the side of the PVC liner every 7-14 cm (sites PC6, PC7, PC8 and PC9). Then we attach copper tubes to the liner as fast as possible. Finally, we may squeeze sediment samples to copper tubes one by one.

**
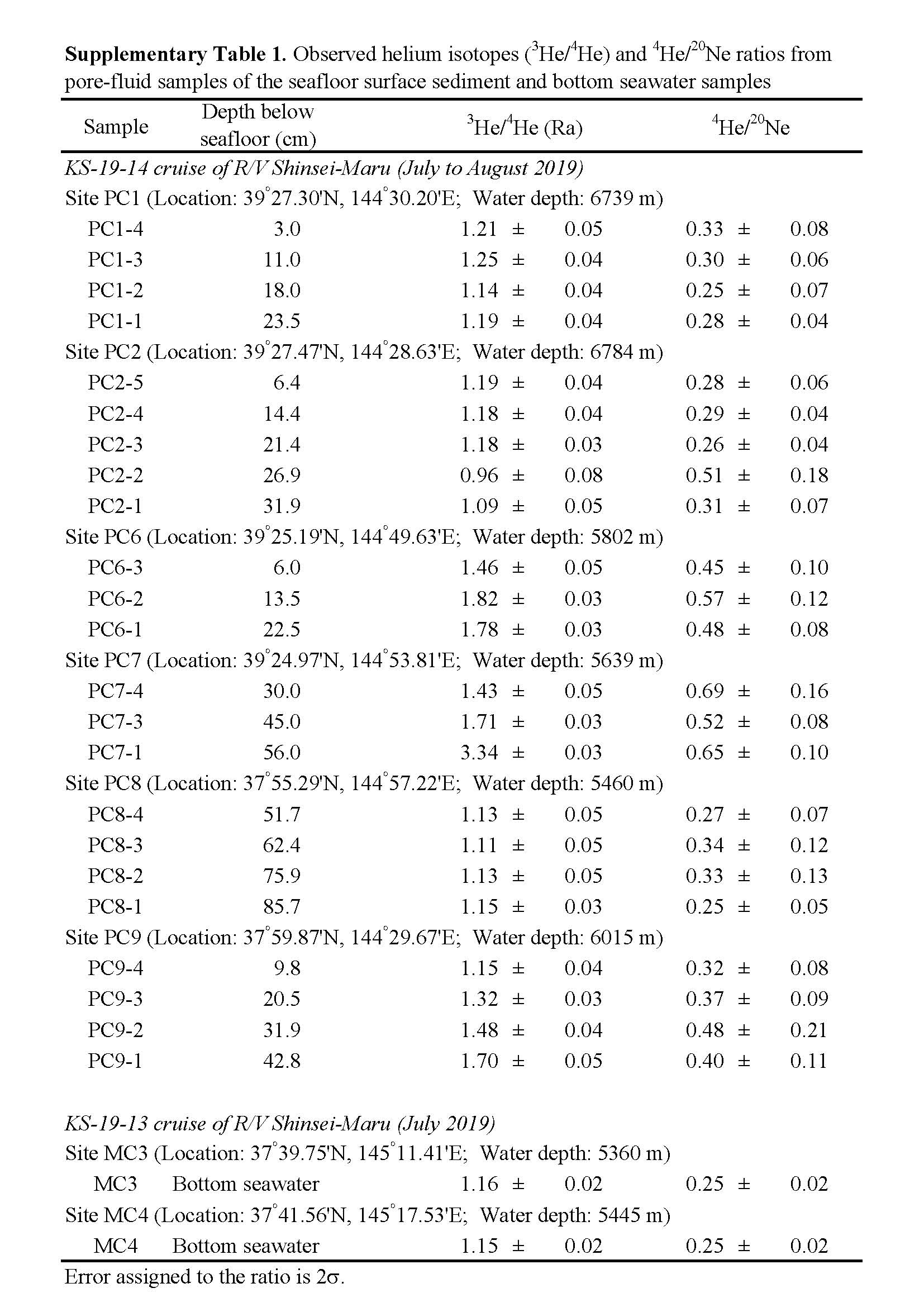
**


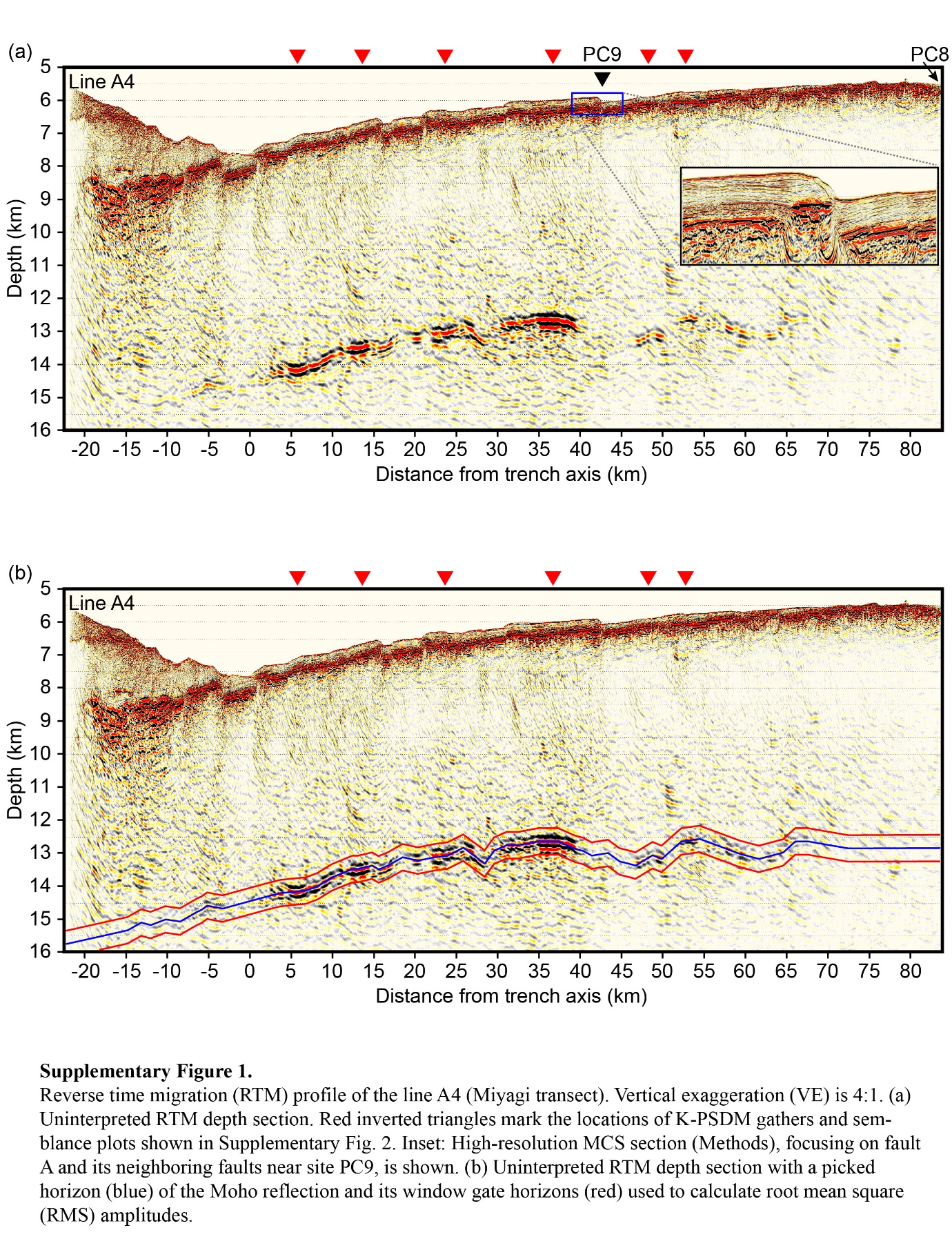


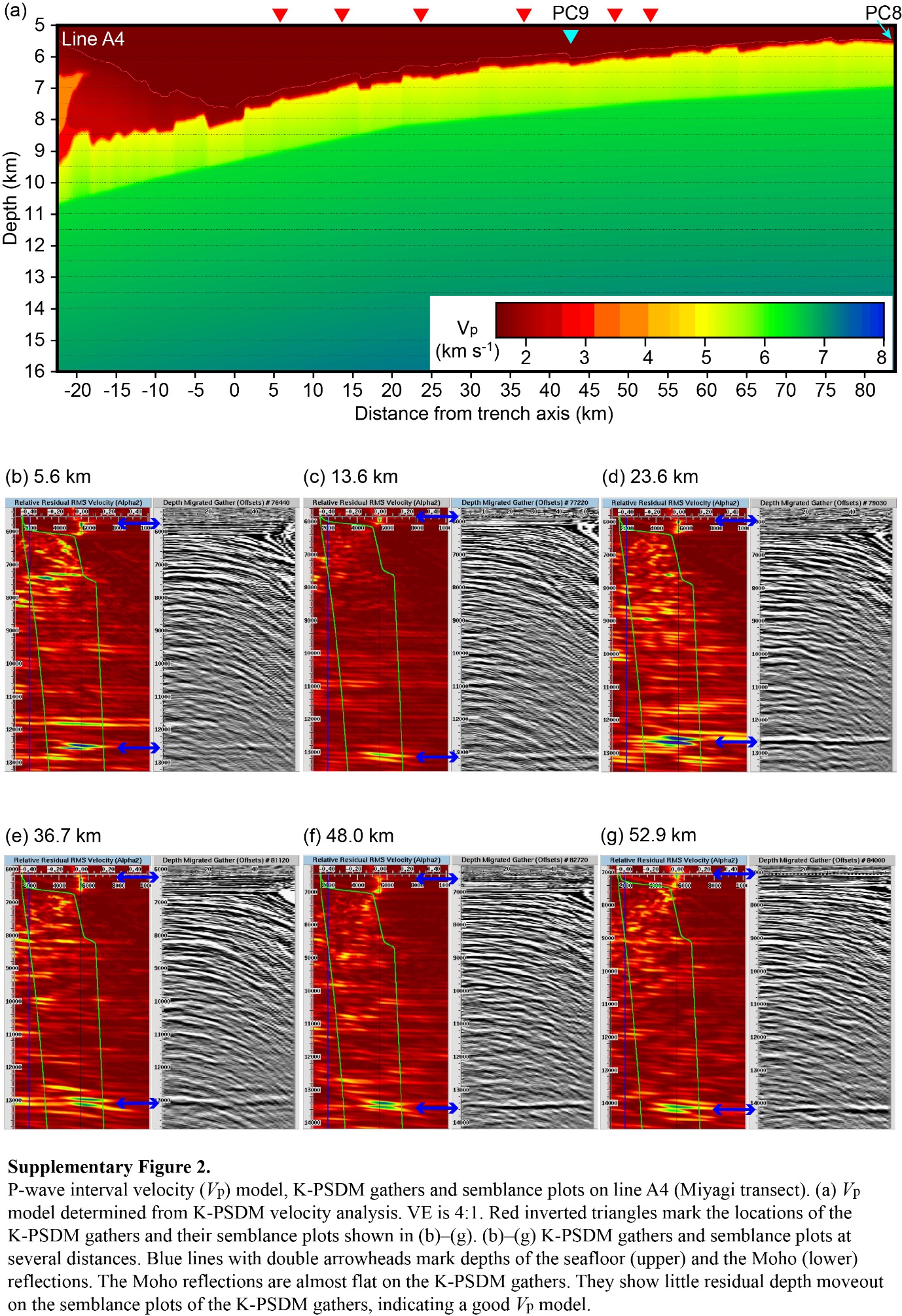


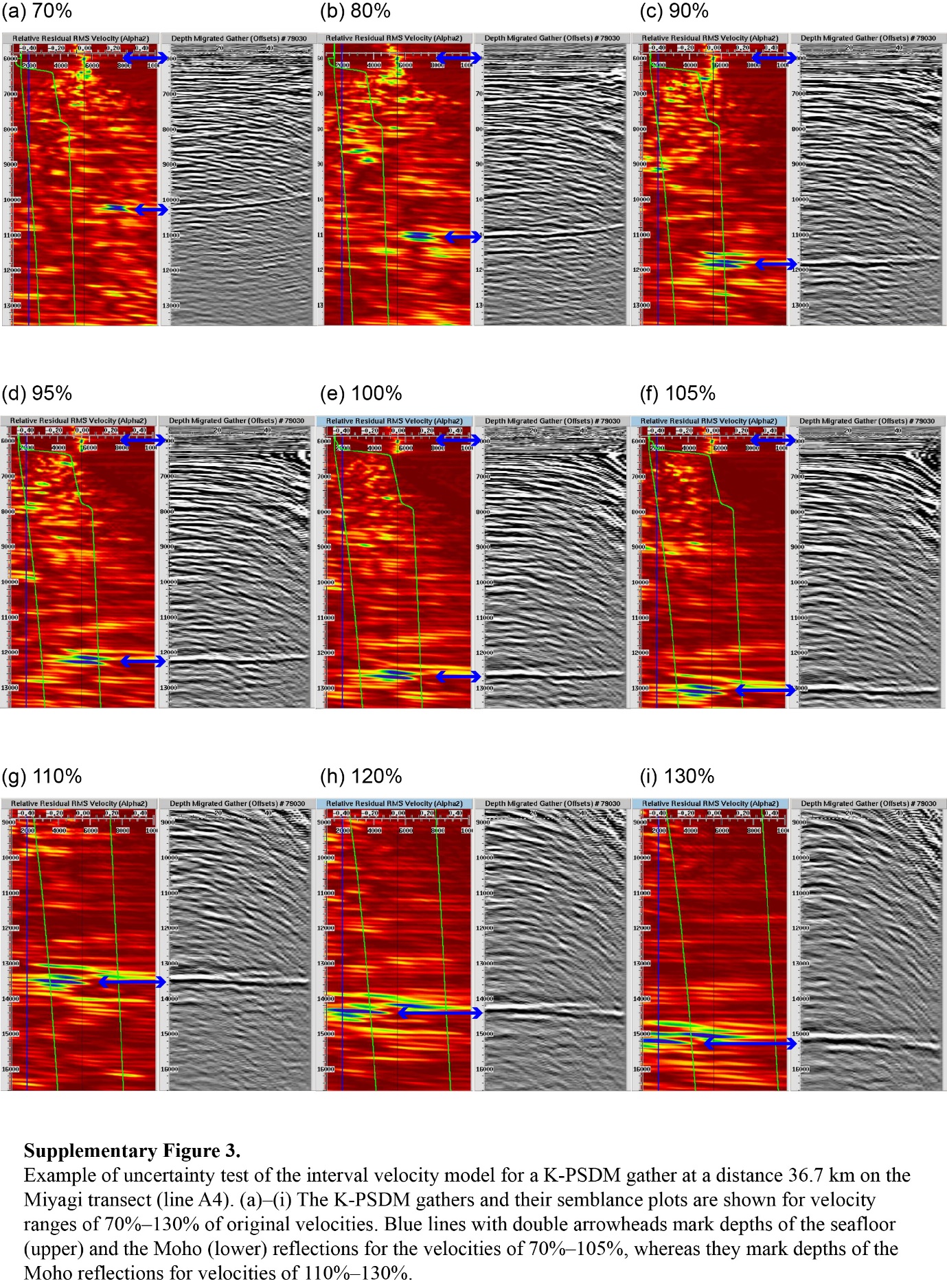


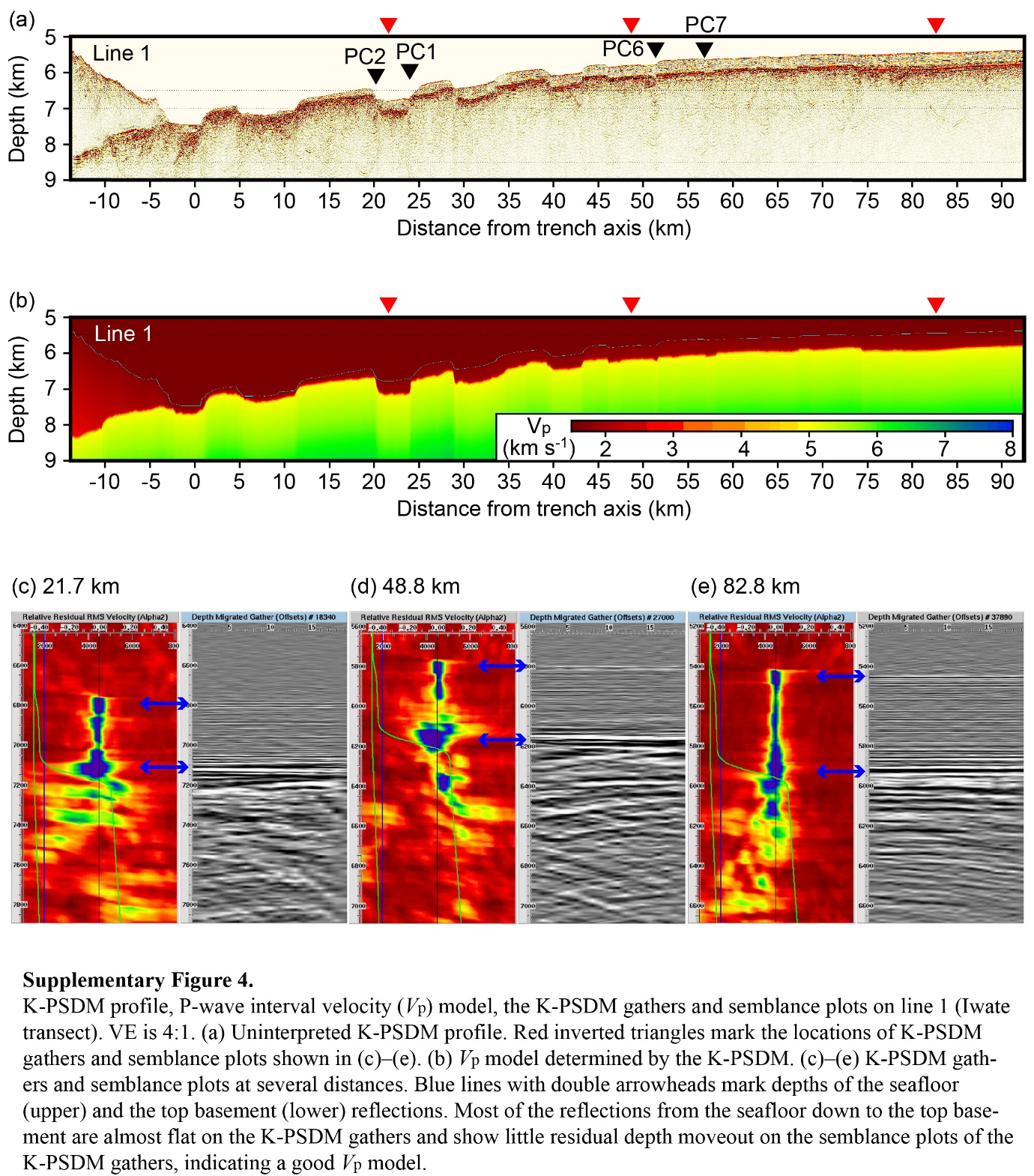


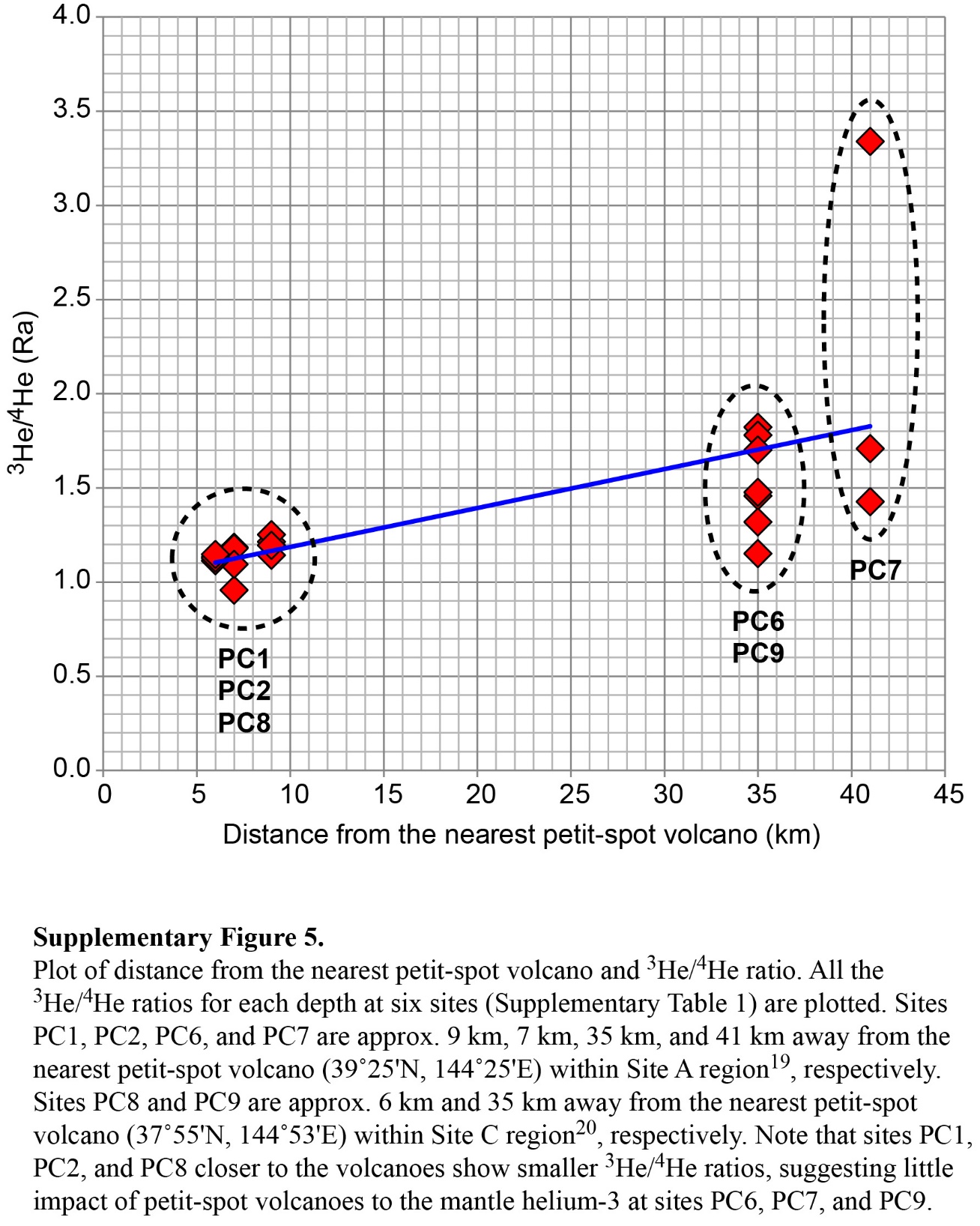

Supplement: Supplementary file 1 — Supplementary Information. [file 41598_2021_91523_MOESM1_ESM.docx]
